# A Nutraceutical Containing Chlorogenic Acid and Luteolin Improves Cardiometabolic Parameters in Subjects with Pre-obesity: A 6-Month Randomized, Double-Blind, Placebo-Controlled Study

**DOI:** 10.3390/nu15020462

**Published:** 2023-01-16

**Authors:** Simona Terzo, Antonella Amato, Antonio Magán-Fernández, Giuseppa Castellino, Pasquale Calvi, Roberta Chianetta, Rosaria V. Giglio, Angelo M. Patti, Dragana Nikolic, Alberto Firenze, Flavia Mulè, Marcello Ciaccio, Manfredi Rizzo

**Affiliations:** 1 Department of Biological, Chemical and Pharmaceutical Sciences and Technologies (STEBICEF), University of Palermo, 90100 Palermo, Italy; 2 Department of Health Promotion, Mother and Child Care, Internal Medicine and Medical Specialties (PROMISE), University of Palermo, 90100 Palermo, Italy; 3 Department of Biomedicine, Neuroscience, and Advanced Diagnostics, Institute of Clinical Biochemistry, Clinical Molecular Medicine, and Laboratory Medicine, University of Palermo, 90100 Palermo, Italy

**Keywords:** pre-obesity, chlorogenic acid, luteolin, cardiometabolic parameters

## Abstract

Pre-obesity is a condition that predisposes to the risk of developing obesity, cardiovascular diseases (CVD), and diabetes. Our previous study demonstrated that a Cynara cardunculus (L.) based nutraceutical named Altilix^®^ (Bionap, Italy), containing chlorogenic acid and luteolin extracts, was able to improve several hepatic and cardio-metabolic parameters. Given this background, we conducted a post-hoc analysis of the Altilix^®^ study in order to analyze the supplement’s effects in the subgroup of pre-obesity subjects on anthropometry (weight and waist circumference), glucose metabolism (HbA1C, HOMA-IR, and HOMA-β), lipid profile (total cholesterol, triglycerides, LDL-cholesterol and HDL-cholesterol), hepatic functionality (FLI, AST, ALT and AST/ALT), carotid-media thickness (CIMT) and endothelial function (FMD). Fifty subjects from the original study cohort (which consisted of 100 subjects) were chosen with BMI ≥ 25 and < 30 kg/m^2^. All subjects received the Altilix^®^ supplement (150 mg/day) or placebo using a computer-based random allocation system. After six months of treatment Altilix^®^ significantly reduced body weight, glycemic, and lipid parameters (total cholesterol, triglycerides, LDL-cholesterol) and improved hepatic functionality, CIMT, and FMD. In conclusion, these results confirm that Altilix^®^ supplementation has a significant effect on cardiometabolic parameters not only in obese subjects but also in pre-obesity subjects.

## 1. Introduction

Obesity is a significant CVD risk factor and the BMI cut-off point to define obesity is 30 kg/m^2^ [1]. In addition to CVD, the pre-obesity condition predisposes toward other complications, such as the risk for cancer that begins to increase at a BMI of 21 kg/m^2^, the risk for diabetes that starts to rise at 22 kg/m^2^, and the risk of musculoskeletal-related illnesses that starts to increase at 24 kg/m^2^. According to data from the Global Burden of Disease 2015 Obesity Collaborators, about 39% of cause-specific deaths occur in people with pre-obesity [2].

Visceral fat accumulation is the first manifestation of pre-obesity status. Adipose tissue is not only an energy-storage organ, but it produces and secretes a variety of biologically active molecules known as adipocytokines. Dysregulation of adipocytokines caused by visceral fat accumulation is regarded as one of the major pathophysiological mechanisms of atherosclerosis associated with metabolic syndrome [3,4]. Because the syndrome occurs commonly in pre-obesity individuals, the risk of developing an atheroma is high. Accordingly, subjects with pre-obesity may have elevated plasma fibrinogen levels, exaggerated platelet activity, reduced fibrinolytic capacity, and increased leptin and C-reactive protein levels, indicating a possible role in the pathogenesis of atherosclerosis [4,5]. Then, ‘lifestyle modification’ via a combination of a healthy diet, physical activity, and behavior therapy is the cornerstone to preventing the pathophysiological defect seen in pre-obesity.

In this context, functional foods and nutraceuticals have a wide range of applications. It is widely recognized that, due to the antioxidant and anti-inflammatory properties of many natural substances, chronic consumption of nutraceuticals in every-day clinical practice can improve the outcome in patients with atherosclerosis, type 2 diabetes mellitus, obesity, and dyslipidemia [6,7,8,9,10]. Furthermore, evidence supports the hypothesis that multiple natural product combinations may result in a synergistic activity that increases their bioavailability and action on multiple molecular targets, offering considerable advantages over a single consumption [11,12]. In our previous randomized, double-blind, placebo-controlled study, a *Cynara cardunculus L.* based nutraceutical named Altilix^®^ (Bionap, Italy) containing, among others, chlorogenic acid and luteolin, with excellent anti-inflammatory and hepatoprotective properties [13], was able to improve several hepatic and cardio-metabolic parameters [14].

On the other hand, a study has demonstrated the efficacy of Cynara extract (600 mg/day) already in overweight subjects with impaired fasting glycemia by reducing glycometabolic parameters [15], pushing us to continue the investigation of this supplement. This study represents a further investigation of the protective effects of Altilix^®^ in the pre-obesity condition. For this purpose, we conducted a post-hoc analysis of the Altilix^®^ study [14] in order to analyze the supplement’s effects in the subgroup of non-obese subjects on anthropometry (weight and waist circumference), glucose metabolism (HbA1C, HOMA-IR and HOMA-β), lipid profile (total cholesterol, triglycerides, LDL-cholesterol and HDL-cholesterol), hepatic functionality (Fatty Liver Index, AST, ALT and AST/ALT), carotid-media thickness (CIMT) and endothelial function (FMD). All subjects included in the present analysis had pre-obesity.

## 2. Materials and Methods

### 2.1. Study Design

**Figure 1:**
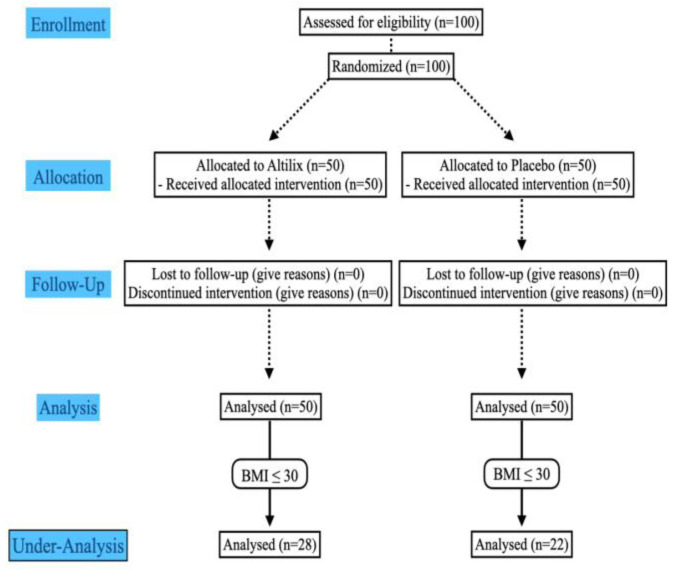
Study design.

This report is a secondary analysis of a randomized, double-blind, placebo-controlled study that demonstrated the ability of the supplement Altilix^®^ to strongly ameliorate several cardiometabolic and hepatic alterations [14]. In the present study, we performed a secondary analysis to verify the effects of the supplement consumption only in subjects with pre-obesity condition. Participant eligibility for this secondary analysis was the same as that for the main trial, except for one parameter: subjects were included if their BMI was ≥25 and <30 kg/m^2^ (Figure 1). Therefore, fifty subjects from the original study cohort (which consisted of 100 subjects) were excluded because they had BMI values above 30 kg/m^2^.

Both nutraceutical and placebo pills were provided by Bionap S.R.L. (Belpasso, Catania, Italy) and were identical in size, color, and consistency to ensure blinding of the participants. The supplement (active ingredients: 10–12% chlorogenic acid and derivates and 2–4% luteolin-7-glucoside and derivates) was obtained from the leaves of *Cynara cardunculus* L. var. *altilis* DC. and *scolymus*. The nutraceutical was prescribed at a stable dose of 150 mg/day for 6 months. Every month, subjects were contacted by phone calls in order to control and reinforce adherence to the study.

The study was conducted according to the Declaration of Helsinki in Italy at the Unit of Diabetes and Cardiovascular Prevention, University Hospital of Palermo for 6 months, and was registered at clinicaltrials.gov (NCT03444558). The study was approved by the Research Ethics Committee of the University Hospital of Palermo (approval number 05/2017 on 10 May 2017). All subjects included in the study provided written informed consent. All subjects received the Altilix^®^ supplement (150 mg/day) or placebo using a computer-based random allocation system. Subjects and investigators were blinded to treatment allocation.

### 2.2. Subjects Characteristics

Subjects were included if they were adults (aged > 18 years) and excluded if they met any of the following criteria: excessive alcohol use, pregnancy, severe hepatic or kidney disease, serious infections, and malignancies. Moreover, all subjects had three or more of the following altered parameters: (1) elevated waist circumference (≥94 cm for men and ≥80 cm for women); (2) elevated triglycerides ≥150 mg/dL (1.7 mmol/L) (or drug treatment); (3) reduced high-density lipoprotein-cholesterol (HDL-C) <40 mg/dL (1.0 mmol/L) for men and <50 mg/dL (1.3 mmol/L) for women (or drug treatment); (4) elevated blood pressure systolic ≥130 and/or diastolic ≥85 mm Hg (or antihypertensive drug treatment); and (5) elevated fasting glucose ≥100 mg/dL (or drug treatment). During the study, any dosage from concomitant therapies remained unchanged in order to avoid potential confounders. All subjects were naïve to statin therapy.

### 2.3. Clinical and Biochemical Evaluation

At baseline and after 6 months of treatments, subjects underwent clinical assessments and laboratory tests, including body weight, waist circumference, systolic blood pressure (SBP), diastolic blood pressure (DBP), ultrasound examination of the carotid arteries and endothelial function. Fasting blood glucose was collected and fasting glycemia, glycosylated hemoglobin, total cholesterol (TC), triglycerides (TG), high-density lipoprotein cholesterol (HDL-C), low-density lipoprotein cholesterol (LDL-C), were measured. Renal function was assessed by serum creatinine levels and estimation of the glomerular filtration rate was performed. Hepatic enzymes alanine aminotransferase (ALT), aspartate aminotransferase (AST), and γ-glutamyltransferase (GGT) were also measured. The AST/ALT ratio was calculated as the ratio between the concentrations of the enzymes AST and ALT. The fatty liver index (FLI) was used as a marker of NAFLD, which was calculated as previously described [12].

### 2.4. Statistical Analysis

Statistical analysis was performed using Stata 14 (StataCorp LLC, College Station, TX, USA). Baseline characteristics were compared using a Student’s t-test and a chi-square test. A paired t-test was used to assess changes of the studied variables within each group. A Student’s t-test was conducted to compare differences in all variables between groups after the follow-up period. A Pearson correlation analysis was performed between the studied variables.

## 3. Results

### 3.1. Characteristics of the Study Population

Of the 100 subjects screened for eligibility, 50 met the inclusion criteria and were recruited in our study. Of these, 28 received Altilix^®^ 150 mg/day and 22 received placebo; in total, 100% of the study population completed the study (Figure 1). Regarding the safety profile, no major adverse events occurred during the study; only two subjects in the treatment group and three subjects in the placebo group referred new-onset transient gastrointestinal symptoms.

### 3.2. Changes in Anthropometric, Metabolic and Cardiovascular Profiles

Anthropometric, metabolic and cardiovascular characteristics of participants divided by Altilix group and placebo group are presented in Table 1.

**Figure 2:**
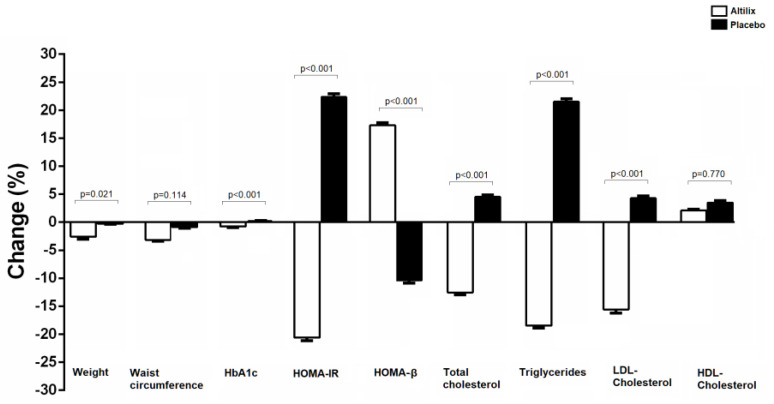
Percentage changes of anthropometric, glycemic and lipid parameters in all subjects.

After six months of treatment, we found a significant reduction in body weight but not in waist circumference in the Altilix group compared to placebo. All glycemic and lipid parameters showed a significant improvement in the Altilix group compared to placebo, with the only exception being HDL-cholesterol. The percentage changes of anthropometric, glycemic and lipid parameters in both groups receiving Altilix and placebo are shown in Figure 2.

**Figure 3:**
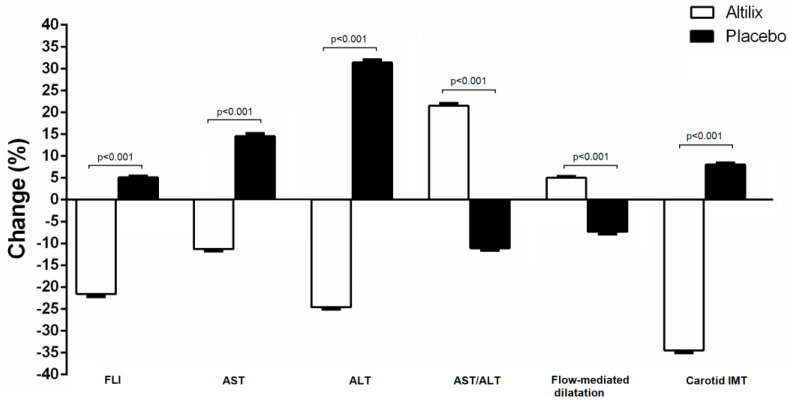
Percentage changes of liver and cardiovascular parameters in all subjects.

As shown in Figure 3, we found at the end of the study a reduction in FLI for those subjects receiving Altilix^®^. By contrast, subjects receiving the placebo had a negative effect. Regarding the liver enzymes, Figure 3 shows the percentage of changes from baseline to 6 months. Both liver enzymes significantly decreased during the treatment, and the reduction was particularly evident for the ALT enzyme, which was reduced by 25% by Altilix^®^. As a consequence, the ratio AST/ALT significantly improved in subjects treated with Altilix compared to placebo. Figure 3 also describes the percentage changes in cardiovascular parameters, namely FMD and carotid IMT. In the Altilix group, we found that FMD increased significantly. FMD increased by 5.4% in the Altilix group, while we observed a reduction by 8% in the placebo group. Compared to the placebo, carotid IMT had decreased at 6 months in the Altilix group, while it increased with placebo (Figure 3).

## 4. Discussion

Previously, we analyzed the effects of the nutraceutical Altilix^®^ containing a combination of chlorogenic acid and its derivatives, and luteolin and its derivatives. We demonstrated that Altilix^®^ could be a valid and safe approach to the prevention and management of cardiometabolic and hepatic alterations. The results pushed us to continue the investigation by performing the present study, a sub-analysis of the Altilix study in which the examination is of subjects with the pre-obesity condition. Here, the use of Altilix improved glycemic and lipid metabolism, liver parameters, and vascular function in pre-obesity individuals. Since this clinical study has had a very rigorous randomized, placebo-controlled, double-blind design, our results emphasize the role of nutraceuticals with proven efficacy in daily practice on subjects with pre-obesity [16]. Our findings are also in agreement with a published report in which supplementation with chlorogenic acid improved the absorption and utilization of glucose from the diet and reduced body mass and body fat in healthy and overweight subjects [17]. Another study demonstrated the health-promoting properties of Luteolin in attenuating adipose tissue inflammation and insulin resistance in HFD mice with loss of ovarian function, suppressing M1-like polarization of macrophages in adipose tissue [18].

Firstly, we analyzed and compared anthropometric variables among the subjects included in the study, as these parameters serve as basic components of validated algorithms for estimating the risk of cardiovascular and hepatic diseases. In our study, waist circumference and body weight decreased after 6 months in the Altilix group versus placebo, although the statistical significance was reached only for the latter. These results are in agreement with another study where overweight subjects with impaired fasting glycemia consuming an extract from *Cynara scolymus* exhibited a significant decrease in anthropometric parameters [15]. The management of body weight by Altilix^®^ is relevant, considering that body weight is associated with cardiometabolic complications, including insulin resistance, metabolic syndrome components, type 2 diabetes, and cardiovascular disease. It is interesting that the weight reduction occurred without any strict adherence to specific low-calorie diets. Indeed, patients were asked to maintain their eating habits, which were characterized by a typical Mediterranean diet.

Alongside the weight maintenance properties of Altilix, beneficial effects on glucose metabolism were also observed in the treated group compared to placebo. Indeed, in the present study, 6 months of Altilix^®^ supplementation reduced HbA1c and improved insulin resistance and beta-cell function. Insulin resistance is characterized by a decline in the cellular response to insulin stimulation in the peripheral tissues and one of the causes is abnormal endogenous ROS production due to excessive fat intake. Antioxidants have been shown to reverse insulin resistance in overweight and obese subjects [19]. Because *Cynara cardunculus* contains multiple antioxidants [20], it likely participates in improving systemic insulin sensitivity by reducing the oxidative stress caused by the pre-obesity condition.

The three major components of dyslipidemia in the pre-obesity condition are increased triglyceride levels, decreased HDL-cholesterol, and changes in the composition of LDL-cholesterol. Since weight loss and improved insulin resistance are linked to an ameliorated dyslipidemia, we also tested the beneficial effects of Altilix against altered plasma lipid levels. On the other hand, our previous report showed that Altilix was able to reduce plasma lipid levels [12,14]. In this secondary analysis, we confirm that the improvement in lipid profile induced by Altilix^®^ is also possible in pre-obesity subjects, as there was a significant reduction in total cholesterol, LDL cholesterol, and triglyceride concentrations in the pre-obesity group compared to placebo. We can speculate that the strong antilipidemic actions of Altilix^®^ are related to chlorogenic acid, the main phenolic compound in the formulation. In fact, chlorogenic acid has exhibited numerous notable metabolic actions in humans and animals, independent of its antioxidant effects [21]. In particular, chlorogenic acid, probably through its interaction with a surface receptor, exerts a set of metabolic actions such as the activation of phosphorylating enzyme 5′ adenosine monophosphate-activated protein kinase (AMPK), a key metabolic sensor [22,23]. AMPK, through post-translational modification, controls the expression of the peroxisome proliferator-activated receptor gamma (PPARγ), a transcription factor related to energy metabolism. Among many functions, this molecule intervenes in the process of lipid β-oxidation and can induce mitochondrial biogenesis [24,25].

Increased levels of hepatic enzymes ALT and AST can be common in pre-obesity, and their prevalence increases progressively with increasing BMI and the dyslipidemia condition. Liver enzyme levels tracked from childhood to adulthood are associated with an adverse cardiovascular profile in adulthood. Therefore, in the present investigation, we verified that he nutraceutical consumption is able to also counteract adverse metabolic conditions occurring in the liver of individuals with pre-obesity. Our results showed that the consumption of Altilix^®^ compared with placebo resulted in a significant reduction in serum concentration of ALT and AST, and an enhancement of the AST/ALT ratio. These findings were consistent with previous research findings. Panahi et al. reported that supplementation with 600 mg of artichoke leaf extract for 2 months significantly decreased serum levels of ALT and AST, improved AST/ALT ratio and APRI scores, and reduced total bilirubin compared to the placebo [26]. Another study conducted by Tang et al. has highlighted a protective effect of artichoke components against acute alcohol-induced liver injury in mice, by showing that an artichoke ethanolic extract significantly decreased AST and ALT in the injured liver. Specifically, treatment with artichoke extract significantly decreased aspartate aminotransferase and alanine aminotransferase, showing a protective effect against acute alcohol-induced liver injury [27]. The protective effects of these supplements were observed in another important index of liver disease, the fatty liver index (FLI), which helps to diagnose fatty liver based on BMI, triglyceride, Gamma Glutamyl Transferase, and waist circumference. FLI is used to identify people at high risk of liver damage without an imaging examination. The FLI ranges are from 1 to 100. A FLI < 30 may be used to rule out NAFLD and a FLI ≥ 60 may indicate the disease [28]. In our study, FLI was significantly reduced in Altilix compared to placebo groups. It can be deduced that the Altilix supplement positively affects the hepatic markers not only in obese subjects but also in pre-obesity subjects. This is consistent with previous findings in animal models on the beneficial effects of nutraceuticals containing chlorogenic acid on NAFLD development and progression [6].

Since a normalization of dyslipidemia, glucose impairment and liver injury could be in turn responsible for atherosclerosis resolution, we wanted to investigate the effects of Altilix on the most used prognosis factors related to the onset and progression of atherosclerosis, such as endothelial functions and carotid IMT. Endothelial function is impaired in obesity and represents the earliest stage of clinical atherosclerosis. Abnormalities in endothelial function worsen with increased weight burden owing to several mechanisms associated with excess fat mass, including impaired glucose tolerance, insulin resistance and metabolic dysregulation, which play a key role in the evolution and clinical expression of cardiovascular disease. Although endothelial dysfunction is a strong predictor of cardiovascular events, restoring arterial homeostasis reduces vascular risk.

In the present study, we showed that dietary Altilix consumption resulted in an increase in flow-mediated dilatation (∼5%) and, thus, an improvement of vascular function. Our findings differ from other observations in which acute administration of chlorogenic acid (400 mg) in healthy subjects did not affect flow-mediated dilatation [29], while they are in accord with another study where 6 weeks of 20 mL/day frozen artichoke juice in moderately hyperlipidemic subjects showed improved vascular function [30]. The reasons for the differences are unlikely to be related to the duration of treatment. Recent improvements in imaging technology have made possible the non-invasive assessment and identification of early vascular changes using ultrasonography. Carotid IMT is constituted by the combined thickness of the intima and media layers of the artery wall, measured at the far wall of the distal common carotid arteries. Carotid IMT is the most widely accepted and the only non-invasive surrogate marker for early atherosclerotic disease [31,32]. Increased carotid IMT is an intermediate stage in the continuum of atherosclerosis, which significantly correlates with coronary and cerebrovascular disease [33,34]. Epidemiological studies have consistently reported a predictive value of increased carotid IMT for myocardial infarction and stroke, independent of traditional CV factors [35,36]. The current study showed a noteworthy decrease in carotid IMT in the experimental group compared with the control group. Luteolin administration was shown to exert beneficial effects against obesity-associated comorbidities, including vascular dysfunction. The effect was attributed to the potent antioxidant and anti-inflammatory characteristics of the polyphenolic flavonoid [37]. Taken together, the metabolic and vascular dysfunction observed in pre-obesity subjects may be improved with the nutraceutical intervention. Therefore, the treatment of pre-obese subjects should be based on a global approach, not only addressing the treatment of insulin resistance and dyslipidemia but also including strategies focused on reducing oxidative stress, inflammation, and cardiovascular risk. In this context, nutraceuticals may have an important role in the prevention of obesity-related metabolic disease, in combination with a healthy diet and lifestyle.

Several explanations may be proposed for our findings, including several mechanisms of antioxidant activity against insulin resistance and the pathogenesis of atherosclerosis. Therefore, we suggest that future studies should focus on investigating the biological mechanism of Altilix^®^, such as its role in oxidative damage/scavenger clearance and in preventing the accumulation of free radicals due to lipid peroxidation. Elucidation of the mechanism of action may lead to a more effective strategy for the prevention of the early metabolic alterations occurring in subjects with pre-obesity conditions. Notably, it is well known that endothelial dysfunction and carotid lesions are strong predictors of future cardiovascular events [38,39] and, therefore, preventive measures, including the use of nutraceuticals with proven benefit and safety [40], are of great clinical importance.

To our knowledge, this is the first randomized, placebo-controlled, double-blind, clinical study evaluating the effect of Altilix^®^ on cardiometabolic risk markers in pre-obesity conditions. Our study has some potential limitations. The main limitation of this study is the missed analysis of Altilix mechanisms of action that does not allow us to demonstrate a cause–effect relationship and, therefore, we can only hypothesize the beneficial role of the main constituents of Altilix^®^ with known anti-oxidant and anti-inflammatory proprieties against metabolic and glycemic alterations. Moreover, an evaluation of inflammatory and oxidative stress biomarkers and adipokines should be performed in future studies. Further research is needed to investigate the effect of Altilix on a broader number of parameters and its mechanism of action. Still, we believe that the main results of the present study are of great clinical importance since we have found that this nutraceutical has a number of beneficial effects on lipid, glucose, hepatic and vascular parameters in pre-obesity subjects, which can be easily translated into the everyday clinical practice of our patients with pre-obesity.

## Figures and Tables

**Table 1:** Anthropometric, metabolic and cardiovascular profile of study participants.

	Altilix (*n* = 28)		Placebo (*n* = 22)		%Change			
	Baseline	6 Months	Baseline	6 Months	Altilix	Placebo	Difference (95% CI)	*p*-Value * (between Groups)
Weight (kg)	73.77 ± 8.23	71.82 ± 8.24	77.68 ± 9.92	77.49 ± 10.62	−2.63	−0.30	−2.33 (−4.30,−0.36)	0.021
Waist circumference (cm)	99.26 ± 8.13	96 ± 7.69	102.9 ± 5.43	102.1 ± 8.06	−3.15	−0.82	−2.33 (−5.23,0.58)	0.114
HbA1c (%)	7.34 ± 1.33	6.6 ± 1.01	7.31 ± 1.78	7.5 ± 1.81	−0.73	0.20	−0.93 (−1.29, −0.57)	<0.001
HOMA-IR	4.18 ± 3.18	3.13 ± 2.02	5.55 ± 5.91	6.24 ± 6.1	−20.8	22.4	−43.17 (−55.69,−30.66)	<0.001
HOMA-β	77.22 ± 52.95	94.54 ± 56.34	78.01 ± 92.25	67.84 ± 80.66	17.3	−10.17	27.49 (15.00,39.98)	0.001
Total cholesterol (mg/dL)	190.21 ± 44.74	166.28 ± 46.77	184.19 ± 36.24	193.14 ± 43.85	−12.6	4.63	−17.25 (−23.29,−11.21)	<0.001
Triglycerides (mg/dL)	168.39 ± 130.32	119.61 ± 51.12	145.52 ± 64.23	175.29 ± 85.98	−18.4	21.6	−40.00 (−55.29, −24.69)	<0.001
LDL- cholesterol (mg/dL)	118.28 ± 37.03	100.78 ± 39.56	108.21 ± 30.69	113.24 ± 35.76	−15.4	4.25	−19.63 (−26.96,−12.30)	<0.001
HDL- cholesterol (mg/dL)	47.93 ± 11.95	48.05 ± 10.63	46.67 ± 10.97	47.84 ± 11.86	2.09	3.44	−1.35 (−10.61,7.9)	0.770
Fatty Liver Index	51.95 ± 23.33	40.73 ± 21.37	58.09 ± 20.76	60.24 ± 21.76	−21.9	5.06	−26.98 (−36.49,−17.47)	<0.001
AST	21.64 ± 9.86	19.61 ± 14.64	20.09 ± 7.58	22.55 ± 7.61	−11.35	14.7	−26.02 (−36.22,−15.82)	<0.001
ALT	28.11 ± 15.91	20.43 ± 11.33	21.59 ± 12.55	27.27 ± 13.21	−24.7	31.5	−56.19 (−66.86, −45.52)	<0.001
AST/ALT	0.843 ± 0.22	0.997 ± 0.28	1.026 ± 0.29	0.914 ± 0.34	21.7	−11.1	32.85 (15.58,50.13)	<0.001
Flow-mediated dilation (%)	20.74 ± 13.3	25.78 ± 13.2	18.26 ± 12.6	10.75 ± 6.97	5.04	−7.51	12.55 (6.67,18.43)	<0.001
Carotid IMT (mm)	0.932 ± 0.16	0.593 ± 0.15	0.819 ± 0.20	0.868 ± 0.23	−34.5	8.09	−42.58 (−55.06,−30.10)	<0.001

All values expressed as mean ± standard deviation and percent change; * Student’s *t*-test.

## Data Availability

Data set is available upon reasonable request.
